# Contribution of Chemical Modifications and Conformational Epitopes to IgE Binding by Ara h 3

**DOI:** 10.3390/foods7110189

**Published:** 2018-11-14

**Authors:** Scott Dyer, Jacqueline B. Nesbit, Beatriz Cabanillas, Hsiaopo Cheng, Barry K. Hurlburt, Soheila J. Maleki

**Affiliations:** 1 Maine General Health-Maine General Allergy and Asthma, Augusta, ME 04330, USA; oxen007@gmail.com; 2 Southern Regional Research Center, Agricultural Research Service, U.S. Department of Agriculture, 1100 Robert E. Lee Blvd, New Orleans, LA 70124, USA; jacqueline.nesbit@usda.gov (J.B.N.); hsiaopo.cheng@usda.gov (H.C.); barry.hurlburt@usda.gov (B.K.H.); 3 Department of Dermatology and Allergy, University of Bonn Medical Center, DE–53127 Bonn, Germany; Beatriz.Cabanillas@ukbonn.de

**Keywords:** peanut, allergy, allergen, processing, structure, conformation, epitope, Ara h 3

## Abstract

Roasting is known to change the allergenic properties of peanuts. To study these observations at a molecular level, the relationship of IgE binding to the structure of Ara h 3 from raw and roasted peanuts was assessed. Ara h 3 (A3) was purified from raw (R), light roast (LR) and dark roast (DR) peanuts, the purity was assessed using sodium dodecyl sulfate-polyacrylamide gel electrophoresis (SDS-PAGE) and the secondary structures were compared with circular dichroism (CD) spectroscopy. In order to understand the contribution of structure to IgE binding, the R A3 was partially denatured (PD) by heat treatment (65 °C for 2 h), subjected to CD spectroscopy and IgE spot blot analysis with sera from peanut- allergic individuals. While we observed that the secondary structure of purified A3 from R and LR peanut in solution was affected by the reduction of disulfide bonds and heat treatment when purified from the peanut following the roasting process, only small alterations were seen in the secondary structure. The purified LR A3 bound higher levels of IgE than the RA3. CD spectroscopy of PD A3 revealed a reduction in the percentage of alpha helices, and serum IgE binding. Therefore, while A3 purified from roasted peanuts did not show significant changes in secondary structure, it showed higher IgE binding than R A3. Therefore, the higher IgE binding to LR A3 was more likely to be due to chemical modifications than structural changes. However, a decrease in the IgE binding was seen if R A3 was deliberately unfolded, indicating that the structure played an important role in IgE binding to A3.

## 1. Introduction

Over 15 million Americans have a food allergy and there is evidence for an 18% increase in prevalence [1,2]. More than half of peanut-allergic individuals have as many as 1–2 accidental ingestions every five years [3]. With the large number of applications for peanut products in processed foods and the potential for cross-contamination of other food products with traces of peanut, it can be very difficult for allergic consumers to avoid accidental ingestions. Therefore, in addition to being a serious public health concern, peanut allergy poses a challenge to the food industry and regulatory agencies in terms of food safety.

Understanding allergens at a molecular level is important for deciphering the immune system-allergen recognition and response, and the cause(s) of allergenicity of foods. The effects of processing on the allergenic potential of food has become increasingly important due to recent studies that have shown that individuals can become more safely desensitized to a food by consuming that food in one processed form versus another [4,5,6,7,8,9]. If the processing-induced changes in allergenic proteins can be determined and correlated with clinical reactivity, designing safer preventative or immunotherapeutic tools via processing methods may be very useful. Previously, we studied the effects of thermal processing on the allergenic properties of peanut proteins [10,11]. Thermal processing can cause multiple non-enzymatic, biochemical reactions to occur in food [12]. One of the best characterized reactions that occurs during thermal processing of foods is known as the Maillard reaction, which has been shown to be important in the development of flavor and color [12]. In addition to protein cross-linking, it is known that Maillard reaction products or advanced glycation end-products (AGEs) [13,14] could lead to the chemical modification of amino acids such as arginine, lysine and cysteine [15]. The changes to protein structure following thermal processing is often due to the heat-induced interactions of sugar components with amino acids to form compounds, such as carboxymethyllysine, malanoidin, and other non-cross-linking and cross-linking modifications to proteins, which are thought to have nutritional, physiological, and toxicological consequences [12,15]. These sugar modifications also render allergens less digestible and can result in larger protein fragments, containing multiple IgE binding sites, to survive digestive enzymes in the gut [16], which are then more capable of cross-linking IgE on the surface of mast cells and causing degranulation. The role of food processing on the allergenic properties of ingested foods has been reported by other studies [10,11,17,18,19,20,21,22,23,24]. Roasting-induced structural changes and chemical mechanisms for increased allergenic properties of the peanut allergens were previously explored in a simulated roasting model [10]. It was found that serum IgE bound Ara h 1 and Ara h 2 at higher levels and the increase in IgE binding was correlated with increased carboxymethyllysine (CML) and other modifications on the surface of the protein [24,25]. Ara h 3, is an 11S seed storage protein, which is highly abundant and makes up ~30% of peanut seed proteins [26]. So far, the IgE binding epitopes have been published for Ara h 3. There are four known linear binding epitopes, with epitope number three having the highest percent recognition, 100%, by peanut allergic patient sera [27]. However, there may be as many as eight binding epitopes [28]. From the linear sequence of Ara h 3, 3D models have been developed [28] and the crystal structure has been solved [29]. It is important to note that it has been shown that monocyte-derived dendritic cells, which are antigen presenting cells, display differential uptake of Ara h 3 purified from raw (R), light roast (LR) and dark roast (DR) peanuts [30]. In this study, we investigated differently processed Ara h 3 at the molecular level. Our aim was to determine if roasting alters the structure and IgE binding properties of Ara h 3, by comparing Ara h 3 purified from raw (RA3), light roast (LRA3) and dark roast (DRA3) peanuts. Additionally, the importance of structure to IgE binding was assessed by comparing IgE binding of partially denatured (PD) or fully denatured (D) RA3 to intact, folded RA3.

## 2. Materials and Methods

### 2.1. Patient Sera

Sera were obtained from the blood of peanut-allergic individuals. The blood was collected after informed consent at Tulane Health Science Center (New Orleans, LA, USA), in accordance with the rules and regulations of the institutional review board. Individual sera from previously well-characterized peanut-allergic patients were used in this study [17]. Briefly, 5 male and 7 female patients aged at the time of blood draw between 4 and 42 years, ImmunoCAP correct to peanut ranged from 0.23–39.3 KU/L and most had peanut specific IgE above 5 KU/L with only one patient <1. Four patients had a history of anaphylaxis with peanut ingestion. Additionally, component testing of 5 of the patient sera with the immuno-solid phase allergen chip (ISAC, Thermo-Fisher, Waltham, MA, USA), which enables testing for specific IgE (sIgE) against multiple allergen components in a multiplex assay, was performed and reported in the Table 1.

### 2.2. Extract Preparation and Protein Purification

Florunner peanuts were used either raw, light roasted, or dark roasted as previously described [19]. Raw, shelled peanuts were roasted in a dry roaster set at 320 °F (160 °C) and removed after 10 min for light (LR) and 30 min for dark (DR) roasting. Ara h 3 was purified from crude peanut extracts (CPEs) by ammonium sulfate precipitation followed by ion-exchange chromatography. To prepare peanut extracts, the peanut was defatted with petroleum ether. Ten grams of defatted CPE was solubilized in 500 mL of buffer (50 mM Tris-Cl, pH 8.3, 5 mM EDTA, 1 mM PMSF) containing 200 mM NaCl. The solution was homogenized with ultrasound treatment on ice at 40% power using a Heat Systems Disrupter. The homogenate was subjected to centrifugation at 13,000 rpm for 30 min at 4 °C. Ara h 3 was precipitated from the cleared homogenate by the addition of a 50% saturated solution of ammonium sulfate while stirring for 30 min on ice. The ammonium sulfate precipitated proteins were collected by centrifugation at 13,000 rpm for 30 min at 4 °C and stored stably at 4 °C. The ammonium sulfate pellet was solubilized in extraction buffer, and then desalted into the same buffer and subjected to Mono-S cation-exchange chromatography (BioRad, Hercules, CA, USA) where the bound Ara h 3 protein was purified by gradient elution with 200 mM–800 mM NaCl in the same buffer to obtain purified Ara h 3 [31].

### 2.3. Circular Dichroism of Purified Ara h 3

Far UV (185–250 nm) circular dichroism (CD) spectra of Ara h 3 purified from raw (R), light roast (LR) and dark roasted (DR) peanuts were obtained. Partially denatured Ara h 3 was obtained by adding either 10 mM dithiothreitol (DTT) for a few minutes or by incubating for 2 h at 65 °C. Denatured Ara h 3 was obtained by heating the sample for 10 min at 95 °C (data not shown) or incubating with 10 mM DTT overnight. Samples were desalted using disposable gel filtration columns (G-25, PD-10, Pharmacia) into Milli-Q water and immediately used in CD measurements or spot blot analysis. A CD spectrum of Milli-Q water was obtained for background purposes. Protein concentrations were 0.1 mg/mL and spectra were obtained at room temperature with a JASCO Model J-815 spectropolarimeter. The mean residue weight of 115.1 g mole^−1^ for Ara h 3 was used for calculating ellipticities. Secondary structural modes were estimated from ellipticities by multiple protein secondary structure prediction and calculation programs such as K2D2 and CDPro.

### 2.4. Sodium Dodecyl Sulfate-Polyacrylamide Gel Electrophoresis (SDS-PAGE)

Raw and roasted samples or purified proteins were subjected to SDS-PAGE on 4–20% Novex Tris-HCl pre-cast gels (Life Technologies, Carlsbad, CA, USA) where individual proteins were separated according to size and stained with Gel-Code Blue (Pierce, Rockford, IL, USA) for 1 h, and digitally recorded with a CCD camera system Fuji-LAS 4000 (Fuji Photo Film Co., Ltd., Duluth, GA, USA).

### 2.5. Spot Blot Analysis of Raw (R), Light Roast (LR) and Partially Denatured (PD) Ara h 3

Different amounts of protein from the pure R, LR, PD and D Ara h 3 samples were desalted into water with a Bio-spin^®^6 (BioRad, Hurcules, CA, USA) according to manufacturer’s instructions, after any denaturing treatment were spotted onto a nitrocellulose membrane, 0.20 µm pore size (Bio-Rad, Hercules, CA, USA). The membrane was blocked with 2% (*w*/*v*) non-fat milk in PBS plus 0.5% Tween-20 (PBST) buffer at room temperature for 30 min. The blocked membranes were then incubated overnight at 4 °C with specific human sera from peanut-allergic patients at a 1:10 dilution in PBST. The membranes were washed in PBS and incubated for 30 min at room temperature with a 1:10,000 dilution of rabbit anti-human IgE with 2% non-fat milk in PBST. The membranes were washed with PBS and the signal detected using ECL-plus (Amersham Biosciences Corp, Piscataway, NJ) according to manufacturer’s instructions.

## 3. Results and Discussion

**Figure 1:**
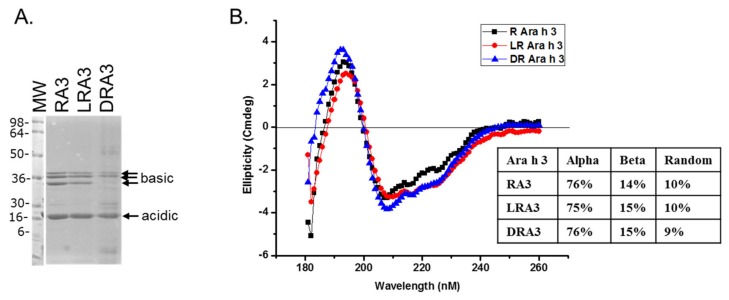
Sodium dodecyl sulfate-polyacrylamide gel electrophoresis (SDS-PAGE) and circular dichroism (CD) spectroscopy of roasted (R), light roast (LR) and dark roast (DR) Ara h 3. (**A**) The protein profile of Ara h 3 purified from, raw, light roast and dark roast peanut; and (**B**) the CD spectrum of each are shown and labeled RA3, LRA3 and DRA3, respectively, reported as ellipticity (on y-axis) as a function of wavelength (nm, x-axis). The percentages of the secondary structure elements are shown in the inset of (**B**).

**Figure 2:**
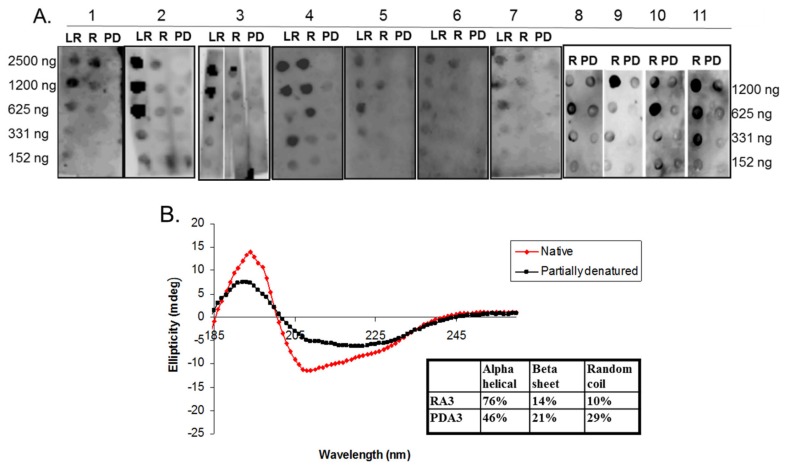
Circular dichroism (CD) spectra and IgE spot blot analysis of varying concentrations of light roast (LR), roasted (R) and partially denatured (PD) Ara h 3 incubated with different peanut allergic sera. (**A**) Equal and decreasing amounts (shown on each side of the blots in nanograms, ng) of each, LRA3, RA3 and PD Ara h 3 (PDA3), are spotted onto nitrocellulose membranes and incubated with individual patients’ sera indicated by number at the top; (**B**) The CD spectra of RA3 and PDA3 reported as ellipticity (on y-axis) as a function of wavelength (nm, x-axis) and the percentages of the secondary structure elements are shown in the inset.

**Figure 3:**
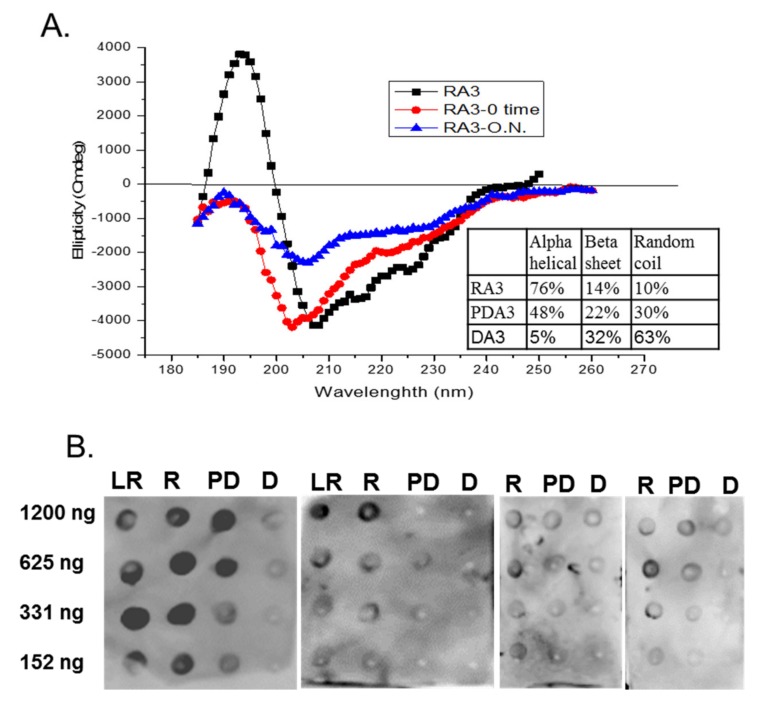
(**A**) Circular dichroism (CD) spectra and IgE spot blots analysis of varying concentrations of light roast (LR), roasted (R), partially denatured (PD) and fully denatured (D) Ara h 3. The percentages of the secondary structure elements are shown in the inset of; (**B**) Equal and decreasing amounts (shown on the left of the blots in nanograms, ng) of each, LR, R, PD and fully denatured (D) Ara h 3, are spotted onto nitrocellulose membranes and incubated with 1:10 dilution of four patients’ sera with history of anaphylaxis to peanut. PDA3 was obtained by incubating with DTT for 5–10 min (0 time, RA-0 time) and DA3 was obtained by incubation with DTT over night (O.N., RA-O.N.).

The belief in the field of protein biochemistry is that heating leads to the loss of structure. Therefore, one of the major peanut allergens, Ara h 3 was purified from raw and roasted peanuts and spotted onto membranes and probed with human IgE from peanut-allergic individuals. We previously made the observation that IgE binding for every serum tested was higher for the roasted than the raw. We theorized that Ara h 3, purified from roasted peanuts, was potentially denatured due to the high temperatures of roasting and this led to exposure of IgE epitopes that were inaccessible in the folded protein of the raw peanut. Therefore, Ara h 3 was purified from raw (R), peanuts and two levels of roasted peanut, light roast (LR) and dark roast (DR) were subjected to SDS-PAGE, and the secondary structure was compared with circular dichroism (CD) spectroscopy (Figure 1A). In this figure the protein migration profiles of the purified, multi-subunit, Ara h 3 are shown and the acidic and basic subunits are present and indicated by arrows. The CD spectral profiles of the R, LR and DR Ara h 3 (referred to as RA3, LA3, DRA3) are displayed as ellipticity (on y-axis). It is important to note that CD data can vary depending on the type of software used and was mostly used for comparative purposes in this study. The data showed that the CD spectra (Figure 1B) were very similar and the percentages of the predicted secondary structural elements did not seem to change significantly with high heat, which indicated that the roasting process, within the compact structure of the peanut, did not cause the Ara h 3 to unfold. Therefore, while some small changes were seen in the structure due to roasting, they did not seem significant enough to account for the differences seen in IgE binding. Therefore, we questioned the importance of structural features or conformational epitopes in IgE binding to Ara h 3. To assess this, we subjected purified RA3 in solution (as opposed to being within the compact structure of peanut) to 65 °C for an hour and compared its IgE binding and structure to the unheated RA3 (Figure 2). Primarily the LR, R and partially denatured (PD) Ara h 3 were spotted onto nitrocellulose membranes at different levels, as indicated in Figure 2A and incubated with individual peanut allergic patient sera (shown by number) overnight. It is important to note that a few of our patient sera had weak IgE binding to Ara h 3, however it was clear that the IgE binding level was highest to lowest to LRA3 > RA3 > PDA3 (Figure 2A). The IgE binding to PDA3 was not eliminated for some patient sera as can be seen in spot blots analysis with sera 8–11 in Figure 2A. This experiment allowed for two important observations. First, as previously observed, the IgE binding was higher to the LRA3 than to the RA3 and therefore, there were some differences between them at a molecular level that resulted in differences in IgE recognition. Meanwhile, as seen in Figure 1, the secondary structures between LRA3 and RA3 were very similar, which led us to speculate that the increased IgE-binding was predominantly, or more likely due to the chemical modifications that occurred within the peanut during roasting, as previously described for Ara h 1 [24], than significant structural alterations. The second observation from these experiments was that once the RA3 partially denatured, losing a significant portion of the alpha helical structure (Figure 2B), the IgE binding was significantly decreased. This finding suggested that the structural components played a significant role in the recognition of Ara h 3 by serum IgE of peanut-allergic individuals. It is well known that IgE binds to linear peptides of Ara h 3, as anywhere from 4–8 IgE epitopes have been identified for the basic subunit of Ara h 3 [27]. IgE binding, while reduced in PDA3, was still visible. In the following experiments, we wanted to determine what effect denaturation of Ara h 3 would have on the IgE binding by patient sera. Methods to denature Ara h 3 with heat and DTT were explored and we found that DTT, as expected, had a significant and near immediate effect on the Ara h 3 structure as can be seen in Figure 3. The Ara h 3 was treated with DTT and then either immediately desalted into water before the CD spectra was measured (0 time point, Figure 3A) or it was spotted onto a membrane for spot blot analysis (Figure 3B). The RA3 was in full random coils after incubation in the presence of DTT overnight, indicated as O.N. in Figure 3A. This sample was also desalted into water for either CD or spot blot analysis with patient IgE (Figure 3B). In Figure 3B, two of the patients’ sera were selected to assess changes in IgE binding between RA3, PDA3 and fully denatured Ara h 3 (DA3). DA3 was shown to have significantly less IgE binding properties, confirming that Ara h 3 partially or fully denatured with a different method (DTT as opposed to heating in solution), lost structural components and coincidentally, IgE binding. Therefore, in addition to previously identified linear epitopes, a significant portion of IgE binding to Ara h 3 is likely due to conformational epitopes.

The effects of roasting on IgE binding and allergenic potency of peanut allergens has been studied for over 20 years. A simulated roasting model of heating crude peanut extract (CPE), and the allergens, Ara h 1 and Ara h 2, purified from raw peanuts, in the presence of reducing sugars at 55 °C for 10 days in solution, enhanced IgE binding [10]. In another similar study, the CPE and purified Ara h 1 and Ara h 2 were dried and heated at 145 °C for 20 min in the presence and absence of glucose [32]. With dry roasting, they found that IgE binding to Ara h 1, in the presence of glucose, was significantly reduced, while the capacity of mediator release increased. Meanwhile, both IgE binding and mediator release with Ara h 2 in the presence and absence of glucose was reduced. In a different study, Ara h 1 that was purified from roasted peanuts was shown to bind higher levels of IgE than Ara h 1 purified from raw peanuts [17]. It was determined that chemical modifications, such as the specific Maillard reaction products, identified on the surface amino acids of the roasted Ara h 1, contributed more to increased IgE binding than major structural alterations [17,24]. Additionally, in one study, AGE modifications were identified on Ara h 1 and Ara h 3 in raw and roasted peanut extract, but not on Ara h 2. We have attributed discrepancies such as these to different methods of experimentation such as protein extraction (time, temperature, buffers), purification, methods of glycation, temperatures of heating, etc. For example, we have found that if purified proteins are heated in the presence or absence of sugar at high temperatures for even short periods of time, depending on the temperature, it is as if the protein or the food extract has been burned or charred, where all of the protein becomes insoluble. Therefore, any subsequent findings are not likely to apply to the actual ingested form [19]. In the present study, we chose to assess Ara h 3 that was roasted within the context of peanuts, and then purified, in order to determine if the protein maintains its structure following the high temperature of roasting. We found that while there were small, observable changes in the secondary structure between the RA3, LRA3 and DRA3, for the most part, the protein secondary structure was maintained. Given the high temperature of roasting, this result might seem surprising. However, the idea that the proteins are packed tightly within the compact structure of the peanut, with limited freedom of movement and solvent accessibility, makes this observation somewhat expected. Therefore, merely purifying a protein from the context of a food and heating it in solution is not representative of the actual thermal processing treatment. We have previously found that IgE binding to roasted peanuts is higher than raw peanuts [10] and here we demonstrated that Ara h 3 from roasted peanuts binds higher IgE than raw Ara h 3 and attribute most of this to the chemical modifications that occur as a result of roasting rather than structural changes. This leads one to question the role of structure in IgE binding. In order to assess the role of structure, we compared the IgE binding properties of RA3 with PDA3 that was heat denatured at 65 °C in solution. A significant decrease was seen in IgE binding to PDA3 compared to RA3 for all patient sera tested, indicating that structure was important in IgE recognition of Ara h 3. A second denaturing method (reduction with DTT) demonstrated the same thing. This emphasized that Ara h 3 contains conformational epitopes that are important for IgE binding. These findings do not obviate the existence of linear epitopes in Ara h 3 that have been previously reported. Our assay system indicates reduction in IgE binding and is not sensitive enough to detect the linear epitope binding by IgE. 

## 4. Conclusions

While model systems can be highly effective in understanding molecular events, it is important to understand the effects of food processing on allergenicity and on the individual allergens within the context of a food in order to develop useful tools for research, diagnosis, detection and preventative or therapeutic treatments. In this study, we demonstrated that the methodology in assessing an allergen’s structural and immunological properties could influence the outcome of a study. We were able to partially denature Ara h 3 at 65 °C and fully denature Ara h 3 at 95 °C (data not shown). Additionally, as expected, disruption of disulfide bonds allowed partial and full denaturation of this protein. However, when Ara h 3 was subjected to the commercial roasting process of 160 °C temperature for 40–60 min, within the context of peanuts, it maintained its structural elements and indeed bound higher levels of IgE then the Ara h 3 purified from raw peanuts. Based on this study, we conclude that increased IgE binding to roasted Ara h 3 is more likely due to roasting-induced chemical modifications of the protein rather than structural changes. Furthermore, given that unfolding the Ara h 3 purified from raw peanuts resulted in significant reductions in IgE binding, we believe that a significant portion of IgE binding to this protein is due to the existence of conformational epitopes.

## Figures and Tables

**Table 1:** Specific IgE levels against Ara h 3 determined by ISAC microarray.

	ISAC Values for Ara h 3 (Fluorescence Intensity)				
Patient number	1	2	3	4	5
Ara h 3 sIgE	1.57	10.6	1.03	2.61	0.78
